# Expression profiles and polymorphic identification of the *ACSL1* gene and their association with body size traits in Dezhou donkeys

**DOI:** 10.5194/aab-63-377-2020

**Published:** 2020-11-10

**Authors:** Zhenyu Lai, Fei Wu, Zihui Zhou, Mei Li, Yuan Gao, Guijun Yin, Jie Yu, Chuzhao Lei, Ruihua Dang

**Affiliations:** 1 Key Laboratory of Animal Genetics, Breeding and Reproduction of Shaanxi Province, College of Animal Science and Technology, Northwest A&F University, Yangling, Shaanxi Province, 712100, China; 2 National Engineering Research Center for Gelatin-based Traditional Chinese Medicine, Dong-E-E-Jiao Co. Ltd., No.78, E-jiao Street, Done-E Country, Liaocheng, Shandong Province, 252201, China

## Abstract

Finding out the genetic mechanism of growth and development traits and the development of related molecular markers can help improve the breeding of livestock. The long-chain acyl coenzyme A synthase 1 (*ACSL1*) gene plays a major role in lipid synthesis and fatty acid catabolism. However, there are few studies on the *ACSL1* gene polymorphism of Dezhou donkeys. This study analyzed the expression level of the *ACSL1* gene in different tissues of young and adult Dezhou donkeys, as well as association analysis of four gene polymorphic loci in 450 individuals. The results showed that expression levels of the *ACSL1* gene are higher in heart, liver, spleen, lung, renal, gastric and muscle tissues of adult donkeys than in those of young donkeys. In the association analysis between genotype and body size traits, the wild genotype DD at the *ACSL1-1* locus in female and male donkeys was greater than the mutant genotype II (P<0.05); genotype II of *ACSL1-2* was significantly higher than that of DD in withers height, body length, rump width and body weight of male donkeys (P<0.05); and *ACSL1-3* showed a tendency for the wild genotype II to be greater than the mutant genotype DD in female and male donkeys (P<0.05). In addition, among the five haplotype combinations constructed, Hap3Hap3 (II-II-DD-DD) and Hap6Hap6 (DD-II-II-II) haplotype combinations were superior to other haplotype combinations in growth traits, which also indicated that the results of haplotype combination association analysis and genotype association analysis tended to be the same. In conclusion, the results of this study indicate that the polymorphic loci of the *ACSL1* gene can be used as candidate molecular markers for the growth and development of Dezhou donkeys, and provide a theoretical reference for the breeding of Dezhou donkeys.

## 1 Introduction

The Dezhou donkey is one of the large fine donkey breeds in China, belongs to the important animal and poultry genetic resources and is also an important variety in the development of the donkey industry. Growth and development indicators are very important breeding concerns in animal husbandry. However, in the research of domestic animal growth and development, there are very few studies on various local breeds of donkeys. Therefore, it is of great significance to study the genetic basis of donkey growth and development traits.

The long-chain acyl coenzyme A synthase *ACSL* family is composed of five *ACSL* subtypes (*ACSL1*, *ACSL3*, *ACSL4*, *ACSL5* and *ACSL6*) and several fatty acid transporters (Ellis et al., 2010). The five subtypes of the *ACSL* family differ in tissue distribution, organelle location, enzyme kinetics, gene expression, regulation and catalytic substrates (Coleman et al., 2000, 2002; Lewin, 2001; Lewin et al., 2002).

The *ACSL1* gene is a key lipid metabolic enzyme in liver organs, which is mainly stored in the intima of adipose tissue and liver cells (Cao et al., 2018). The study found that *ACSL1* has obvious functions in fat differentiation, while other *ACSL* isozymes could not be achieved in adipose tissue (Ellis et al., 2010). The expression of the *ACSL1* gene in rat myocardium gradually increases with the age of the individual until it reaches the highest value in adulthood (Marra and de Alaniz, 1999). Polymorphism analysis of the *ACSL1* gene in large white pigs revealed a correlation between genotype and back fat thickness (Li, 2015). Analysis of quantitative trait loci (QTL) showed that the *ACSL1* gene is a candidate gene for the location and function of fatty acid composition of bovine skeletal muscle, and may play an important role in regulating the lipid composition of beef (Philipp et al., 2011). Haplotype and genotype association analysis of genetic mutations in the promoter region of the yak *ACSL1* gene has revealed a significant correlation with traits such as milk fat percentage (Zhao et al., 2019). *ACSL1* is a target gene of *PPAR*γ, and its expression in fat and skeletal muscle is induced by *PPARa* and *PPAR*γ. The G74T genetic variation of the seventh exon of the bovine *PPAR*γ gene is correlated with growth traits such as chest circumference and body weight, while the G133C and C170T genetic variation of the second intron is highly significant for growth traits such as chest circumference and body weight (Han, 2017; Li et al., 2012; Zhao, 2016). Through the selective signal analysis of six local breeds including the Dezhou donkey, it was found that 11 selected signal regions are correlated with coat color traits, body size traits, movement traits and plateau adaptability, among which *ACSL4*, *NCAPG* and *TBX3* genes are correlated with body size traits (Fan, 2019). Therefore, it is speculated that the *ACSL1* gene may be related to growth traits of Dezhou donkeys.

So far, the expression level and genetic variation of the *ACSL1* gene have not been reported in Dezhou donkeys. In this research, the expression level of the *ACSL1* gene and the correlation analysis between gene polymorphism and growth traits of the Dezhou donkey were studied, which will provide some theoretical basis for molecular breeding of the Dezhou donkey.

## 2 Materials and methods

### 2.1 Animals and RNA and DNA samples

The study was approved by the ethics committee of Northwest A&F University (Yangling, Shaanxi, China) (approval number: 20171208–010, 8 December 2017). Tissue samples were collected from the heart, liver, spleen, lungs, kidneys, stomach and muscles of three young (2 months old) and three adult (more than 2 years old) Dezhou donkeys. Blood samples were collected from 450 individuals, including 206 females and 244 males in the breeding farm of Dong-e-e-jiao Co. Ltd. RNAiso Plus (Takara) was used to extract total RNA from tissue samples. DNA was extracted from blood samples collected from the jugular vein in accordance with the standard scheme of phenol and chloroform. D260 / D280 values were detected by spectrophotometer, and RNA and DNA samples were stored in a refrigerator at -80${}^{\circ }$C. At the same time, the growth traits of the Dezhou donkey were measured (withers height, body length, chest circumference, chest width, chest depth, cannon circumference, rump length, rump width, rump height and body weight).

### 2.2 qPCR detection, polymorphism identification and genotyping of the *ACSL1* gene

**Figure 1:**
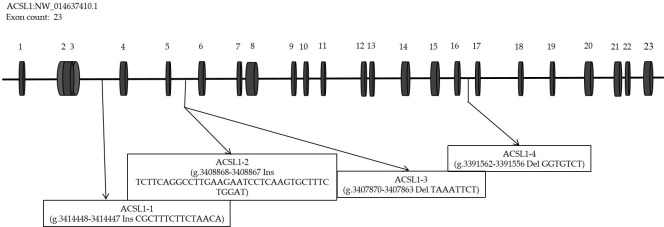
Schematic expression of the *ACSL1* gene and location of identified mutation sites.

According to the analysis of genome resequencing data, there are four potential indel fragments in the intron of the *ACSL1* gene (Fig. 1). Based on the donkey's *ACSL1* and *GAPDH* gene sequence published in the GenBank database (accession number: NW_01463740.1; NW_014638362.1), Primer-BLAST (https://www.ncbi.nlm.nih.gov/tools/primer-blast/, last access: 5 April 2020) was used to design specific primers for quantitative polymerase chain reaction (qPCR) and four mutation sites (Table 1).

**Table 1:** Primer information of Dezhou donkey *ACSL1* and *GAPDH* genes for qPCR and *ACSL1* polymorphic sites.

Loci	Primer sequences (5${}^{\prime }$–3${}^{\prime }$)	Tm (${}^{\circ }$)	Product length	GenBank no.
*ACSL1*	F:CGAAACTGGTGGCTCTGTAATTC	60${}^{\circ }$		NW_014637410.1
(qPCR)	R:CTCGTGGCGTACCAGAAAGT			
*GAPDH*	F:GAGGACCAGGTTGTCTCCTG	60${}^{\circ }$		NW_014638362.1
(qPCR)	R:TCTTGCTGGGTGATTGGTGG			
*ACSL1-1*	F:CATTAGCGCACCAGCATGTC	61${}^{\circ }$	433 + 15 bp	NW_014637410.1
	R:CTAGTGTGCTTGCGGACTCA			
*ACSL1-2*	F:ACCCAGGTGACCAGAGAGAT	62${}^{\circ }$	345 + 38 bp	NW_014637410.1
	R:GGCTCCAGTCAAGTCCAGTT			
*ACSL1-3*	F:CCCCTAGCATTTTGTGATTTTAGAT	62${}^{\circ }$	224 - 8 bp	NW_014637410.1
	R:GAGTAGCAGAGCAGCCAAAAC			
*ACSL1-4*	F:CCCGTCATGCCACGTTAATC	60${}^{\circ }$	181 - 7 bp	NW_014637410.1
	R:GGCCGACTGTGGTGAAATCT			

Tm stands for temperature. + is the insertion base number. - is the deletion base number.

In this study, the product description of AceQ™ qPCR SYBR^®^ Green Master Mix kit (Vazyme, Nanjing, China) was used as a reference for qPCR reaction conditions. The optimized qPCR reaction system is a qPCR amplification system totalling 10 µL, including 5 µL of AceQ™ qPCR SYBR^®^ Green Master Mix, 3.6 µL of RNase-free ddH${}_{2}$O, 1 µL of DNA template, 0.2 µL of upstream primer and 0.2 µL of downstream primer. Three replicates for each individual were performed and amplified according to the following reaction conditions: pre-denaturation at 95 ${}^{\circ }$C for 5 min; cyclic response at 95 ${}^{\circ }$C for 10 s and 60 ${}^{\circ }$C for 30 s, 40 times; and dissolution curve at 94 ${}^{\circ }$C for 30 s, at 60 ${}^{\circ }$C for 1 min and 30 s, and at 94 ${}^{\circ }$C for 10 s. The results after amplification were determined by the dissolution curve and the threshold cycle (Ct) value.

PCR reaction was performed using the 2× Es Taq MasterMix (dye) product description (CWBIO, Jiangsu, China): a total of 12.5 µL of PCR amplification system, including 6.25 µL of 2× Taq PCR Master Mix, 4.75 µL of ddH${}_{2}$O, 0.5 µL of 50 ng µL${}^{-1}$ DNA template, 0.5 µL of upstream primer and 0.5 µL of downstream primer. The amplification was carried out according to the following reaction conditions: pre-denaturation at 95 ${}^{\circ }$C for 5 min; denaturation at 95 ${}^{\circ }$C for 30 s, annealing for 30 s (see Table 1 for annealing temperature of each primer) and extension at 72 ${}^{\circ }$C for 30 s, cycle 32 times; and extension at 72 ${}^{\circ }$C for 10 min. The amplified products were subjected to 10 % polyacrylamide gel electrophoresis detection and sequencing verification.

### 2.3 Statistical analysis

qPCR detection was performed on the *ACSL1* gene and internal reference *GAPDH* gene in different tissues of young and adult Dezhou donkeys, and the obtained quantitative data were calculated and analyzed by the 2${}^{\Delta \Delta -\mathrm{Ct}}$ method (Wu, 2019). The independent sample T test in SPSS 22.0 software (Statistical Product and Service Solutions, Version 22.0, IBM, Armonk, NY, USA) was used to analyze the quantitative results, and P<0.05 was the significant difference. At the same time, GraphPad Prism 7 software was used to draw pictures (Wu, 2019).

The genotype frequency, allelic frequency and Hardy–Weinberg equilibrium (HWE) partial value of four mutant loci in the Dezhou donkeys with different gender were analyzed by the Hardy–Weinberg test software. At the same time, the population genetic parameters were estimated using online software (http://www.msrcall.com/, last access: 5 April 2020), including homozygosity ($H_{o}$), heterozygosity ($H_{e}$), effective number of alleles ($N_{e}$) (Nei, 1974) and polymorphism information content (PIC) (Botstein et al., 1980). The $D^{\prime }$ (LD${}^{\prime }$ coefficient) and $r^{2}$ (correlation coefficient) values between alleles at each point were calculated by SHEsis online software (http://analysis.bio-x.cn/myAnalysis.php, last access: 6 April 2020), and the haplotype frequency between the sites was also calculated (Yong and He, 2005). The correlation between genotype, haplotype combination and growth traits of Dezhou donkeys of different genders was analyzed by one-way ANOVA in SPSS 22.0 software. The statistical model was as follows: Y=μ+e, where Y is the observation of the growth traits, μ is the overall mean of each trait and e represents the random error.

## 3 Results

### 3.1 Comparison of *ACSL1* gene expression in different tissues of Dezhou donkeys of different ages

**Figure 2:**
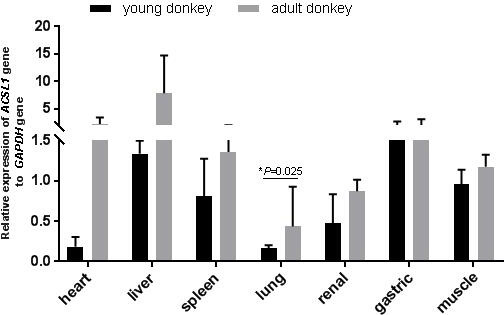
Expression levels of the *ACSL1* gene in tissues of the Dezhou donkey with different stages

In this study, qPCR was used to detect the expression of the *ACSL1* gene in seven tissue samples of the heart, liver, spleen, lungs, kidneys, stomach and muscles of young and adult Dezhou donkeys. As shown in Fig. 2, the expression levels of the *ACSL1* gene were highest for young donkeys in the liver and gastric organs, and highest for adult donkeys in the heart and liver. In addition, the expression of the *ACSL1* gene in seven tissues was higher in adult donkeys than in young donkeys, and the expression in lung tissue of adult donkeys was significantly higher than that of young donkeys (P<0.05), indicating that the expression of the *ACSL1* gene in each tissue increases with age.

### 3.2 Polymorphisms and genetic diversity

**Figure 3:**
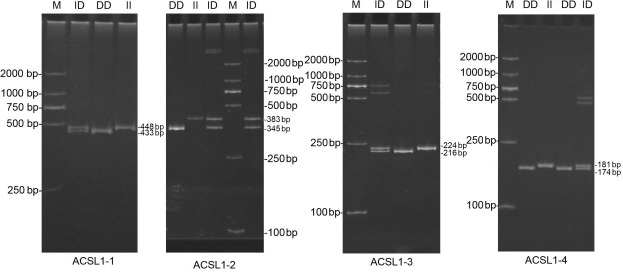
Detection of four mutation sites of the *ACSL1* gene in Dezhou donkeys by PCR. II: insertion/insertion; ID: insertion/deletion; DD: deletion/deletion; and M: marker2000.

**Figure 4:**
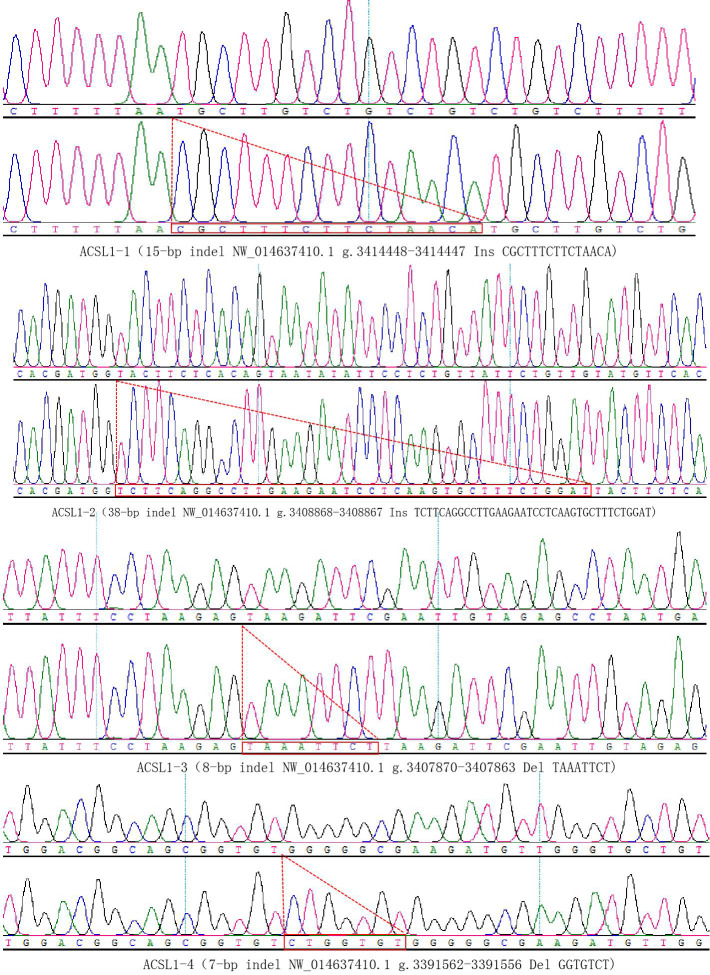
Sequencing validation of four mutation sites of the *ACSL1* gene.

DNA samples of 450 Dezhou donkey individuals were used for PCR amplifying and genotyping by 10 % polyacrylamide gel electrophoresis; the genotyping results are shown in Fig. 3. Three genotypes (II: insertion/insertion; ID: insertion/deletion; and DD: deletion/deletion) were found at all loci. The wild genotype DD of *ACSL1-1* was 433 bp for one fragment, the mutant genotype II was 448 bp for one fragment and the heterozygous genotype ID was two fragments (433 and 448 bp). The wild genotype DD of *ACSL1-2* was one 345 bp fragment, the mutant genotype II was a fragment of 383 bp and the heterozygous genotype ID was two fragments of 345 and 383 bp. At the site of *ACSL1-3*, the wild genotype II was represented by 224 bp fragments, the mutant genotype DD was represented by 216 bp fragments, and the heterozygous genotype ID was represented by 216 and 224 bp fragments. At the site of *ACSL1-4*, the wild genotype II was represented by 181 bp fragments, the mutant genotype DD was represented by 174 bp fragments, and the heterozygous genotype ID was represented by 174 and 181 bp fragments. Some randomly selected individuals were used for sequencing verification; the results are consistent with the electrophoresis genotyping (Fig. 4).

The genotype frequency, allele frequency and genetic diversity parameters ($H_{o}$, $H_{e}$, $N_{e}$ and PIC) of the four *ACSL1* mutation sites are summarized in Table 2. The HWE test showed that the *ACSL1-4* locus was not in HWE in the female donkey population (P<0.05) but was so in male donkeys (P>0.05), while other loci were in HWE in the Dezhou donkey population (P>0.05). In order to identify the genetic diversity parameters of different genders in the Dezhou donkey, the PIC calculations were performed at four loci; the PIC value ranged from 0.234 to 0.375, the *ACSL1-1* locus was in low polymorphism (P<0.25) in female Dezhou donkeys and the other loci were in moderate polymorphism (0.25<P<0.5).

**Table 2:** Detection of genotype frequencies, allele frequencies and population genetic parameters of *ACSL1* gene mutation loci in Dezhou donkeys of different genders.

Loci	Gender	Sizes	Genotypic frequencies (number)			Allelic frequencies		HWE	Population parameters			
		N	II	ID	DD	I	D	P values	$H_{o}$	$H_{e}$	$N_{e}$	PIC
*ACSL1-1*	Female	205	0.702 (n = 144)	0.273 (n = 56)	0.025 (n = 5)	0.839	0.161	0.872	0.729	0.271	1.370	0.234
	Male	241	0.676 (n = 163)	0.291 (n = 70)	0.033 (n = 8)	0.822	0.178	0.885	0.707	0.293	1.415	0.250
*ACSL1-2*	Female	206	0.248 (n = 51)	0.544 (n = 112)	0.208 (n = 43)	0.519	0.481	0.201	0.501	0.499	1.997	0.375
	Male	244	0.271 (n = 66)	0.504 (n = 123)	0.225 (n = 55)	0.523	0.477	0.873	0.501	0.499	1.996	0.375
*ACSL1-3*	Female	205	0.068 (n = 14)	0.420 (n = 86)	0.512 (n = 105)	0.278	0.722	0.520	0.599	0.401	1.671	0.321
	Male	244	0.041 (n = 10)	0.393 (n = 96)	0.566 (n = 138)	0.238	0.762	0.181	0.638	0.362	1.568	0.297
*ACSL1-4*	Female	205	0.171 (n = 35)	0.566 (n = 116)	0.263 (n = 54)	0.454	0.546	0.043	0.504	0.496	1.983	0.373
	Male	244	0.172 (n=42)	0.516 (n = 126)	0.312 (n = 76)	0.430	0.570	0.406	0.510	0.490	1.962	0.370

II: insertion/insertion; ID: insertion/deletion; DD: deletion/deletion; HWE: Hardy–Weinberg equilibrium; $H_{e}$: heterozygosity; $H_{o}$: homozygosity; $N_{e}$: effective allele number; PIC: polymorphism information content; PIC < 0.25: low polymorphism; 0.25 < PIC < 0.5: intermediate polymorphism; and PIC > 0.5: high polymorphism.

### 3.3 Linkage disequilibrium and haplotype analysis of *ACSL1* mutation sites

**Figure 5:**
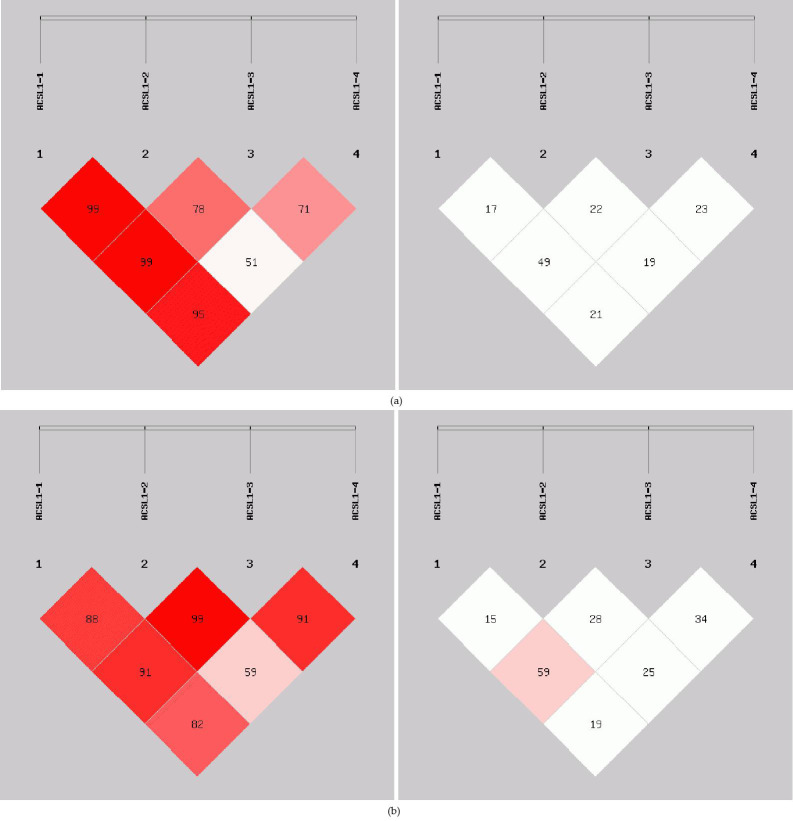
Linkage disequilibrium detection between *ACSL1* mutation sites in Dezhou donkeys. **(a)** Linkage disequilibrium analysis of female donkeys; **(b)** linkage disequilibrium analysis of male donkeys.

In order to reveal the linkage of *ACSL1* gene mutation sites in Dezhou donkeys of different genders, linkage analysis was conducted on the four loci (Fig. 5). Values for $D^{\prime }$ and $r^{2}$ ranged from 0 to 1; $r^{2}>0.33$ is indicative of strong linkage disequilibrium (Kristin et al., 2002). The obtained $r^{2}$ value suggests that there was a strong linkage between *ACSL1-1* and *ACSL1-3* in the female Dezhou donkey population ($r^{2}=0.49$); in the male Dezhou donkey population, there was a strong linkage between *ACSL1-3* and *ACSL1-1* and between *ACSL1-3* and *ACSL1-4* ($r^{2}=0.59$; $r^{2}=0.34$), and other loci were weakly linked ($r^{2}<0.33$).

Haplotype analysis of genetic mutation sites of the *ACSL1* gene (*ACSL1-1*, *ACSL1-2*, *ACSL1-3* and *ACSL1-4*) showed that 6 haplotypes with frequencies greater than 0.05 were identified in female and male donkeys, and were labeled as Hap1–Hap6 (Table 3). Among them, the lowest haplotype frequency (Hap1) and the highest value (Hap5) of female donkeys were 0.082 and 0.347, and the lowest haplotype frequency (Hap1) and the highest value (Hap5) of male donkeys were 0.062 and 0.385, respectively.

**Table 3:** Haplotypes of the four sites within the *ACSL1* gene in Dezhou donkeys of different genders.

Gender	Haplotypes	Loci				Frequencies
		*ACSL1-1*	*ACSL1-2*	*ACSL1-3*	*ACSL1-4*	
Female	Hap1	I	I	I	I	0.082
	Hap2	I	I	D	I	0.107
	Hap3	I	I	D	D	0.161
	Hap4	I	D	D	I	0.106
	Hap5	I	D	D	D	0.347
	Hap6	D	I	I	I	0.158
Male	Hap1	I	I	I	I	0.062
	Hap2	I	I	D	I	0.123
	Hap3	I	I	D	D	0.162
	Hap4	I	D	D	I	0.085
	Hap5	I	D	D	D	0.385
	Hap6	D	I	I	I	0.162

All frequencies <0.05 are ignored in the analysis.

### 3.4 Association analysis of growth traits with genotype and haplotype combinations

The association analysis results between the *ACSL1* gene mutation site and growth traits of Dezhou donkeys are shown in Table 4. The results show that the wild genotype DD at the *ACSL1-1* locus was larger than the mutant genotype II in growth traits, and there were significant differences in withers height, body length, chest depth, rump length, rump width and rump height in female donkeys (P<0.05), and significant differences in chest circumference, chest width, rump width and body weight in male donkeys (P<0.05). At the *ACSL1-2* locus, the genotype II was significantly higher than the DD type in withers height, body length, chest circumference, chest width, chest depth, rump length, rump width, rump height and body weight of male donkeys (P<0.05). *ACSL1-3* also showed a tendency for the wild genotype II to be larger than the mutant genotype DD in terms of growth traits, among which there were significant differences in withers height, chest depth and rump width of female donkeys (P<0.05) and significant correlation with rump width of male donkeys (P<0.05). However, unlike the other three loci, there was no correlation between the *ACSL1-4* locus and growth traits of the Dezhou donkey (P>0.05).

To further verify the relationship between the *ACSL1* gene mutation site and the growth traits of Dezhou donkeys, five homozygous haplotype combinations (Hap1Hap1, Hap3Hap3, Hap4Hap4, Hap5Hap5 and Hap6Hap6) were constructed in female donkeys, and five homozygous haplotype combinations (Hap2Hap2, Hap3Hap3, Hap4Hap4, Hap5Hap5 and Hap6Hap6) in male donkeys. In the haplotype association analysis (Table 5), it was found that the haplotype combination had significant differences in the withers height, body length, rump length and rump height of female donkeys, while there were significant differences in the chest width and rump width of male donkeys. On the whole, Hap3Hap3 (II-II-DD-DD) and Hap6Hap6 (DD-II-II-II) haplotype combinations were superior to other haplotype combinations in growth traits of female and male donkeys, which also indicated that the results of haplotype association analysis and genotype association analysis tended to be the same.

**Table 4:** Association between *ACSL1* gene mutation loci and growth traits in Dezhou donkeys of different genders.

Loci	Gender	Growth traits	Observed genotypes (mean ± SE)			
			II	ID	DD	P values
*ACSL1-1*	Female	withers height	135.00 ± 5.99${}^{\mathrm{ab}}$ (n = 144)	133.55 ± 8.81${}^{b}$ (n = 56)	140.40 ± 7.60${}^{a}$ (n = 5)	0.035
		body length	135.25 ± 6.53${}^{a}$ (n = 144)	133.03 ± 7.58${}^{b}$ (n = 56)	141.00 ± 10.22${}^{a}$ (n = 5)	0.017
		chest depth	56.00 ± 3.05${}^{a}$ (n = 144)	55..00 ± 3.56${}^{b}$ (n = 56)	57.80 ± 4.09${}^{a}$ (n = 5)	0.049
		rump length	43.07 ± 2.53${}^{b}$ (n = 144)	42.46 ± 2.61${}^{b}$ (n = 56)	46.80 ± 4.71${}^{a}$ (n = 5)	0.002
		rump width	43.31 ± 3.40${}^{a}$ (n = 144)	41.83 ± 3.37${}^{b}$ (n = 56)	43.50 ± 3.64${}^{a}$ (n = 5)	0.006
		rump height	137.03 ± 6.25${}^{\mathrm{ab}}$ (n = 144)	135.13 ± 7.11${}^{b}$ (n = 56)	141.6 ± 8.14${}^{a}$ (n = 5)	0.035
	Male	chest circumference	148.23 ± 8.02${}^{b}$ (n = 163)	148.70 ± 9.16${}^{\mathrm{ab}}$ (n = 70)	154.38 ± 5.58${}^{a}$ (n = 8)	0.042
		chest width	33.19 ± 2..18${}^{b}$ (n = 163)	32.94 ± 2.72${}^{a}$ (n = 70)	35.25 ± 1.93${}^{a}$ (n = 8)	0.033
		rump width	40.06 ± 3.03${}^{b}$ (n = 163)	40.37 ± 3.68${}^{b}$ (n = 70)	44.75 ± 3.37${}^{a}$ (n = 8)	0.001
		body weight	290.50 ± 42.86${}^{a}$ (n = 86)	270.95 ± 58.04${}^{b}$ (n = 44)	318.00 ± 39.33${}^{a}$ (n = 6)	0.024
*ACSL1-2*	Male	withers height	138.17 ± 7.02${}^{a}$ (n = 66)	136.25 ± 6.42${}^{\mathrm{ab}}$ (n = 123)	135.63 ± 6.19${}^{b}$ (n = 55)	0.034
		body length	138.45 ± 8.09${}^{a}$ (n = 66)	135.25 ± 7.86${}^{b}$ (n = 123)	133.60 ± 7.16${}^{b}$ (n = 55)	0.002
		chest circumference	151.51 ± 7.78${}^{a}$ (n = 66)	147.72 ± 8.53${}^{b}$ (n = 123)	146.94 ± 7.58${}^{b}$ (n = 55)	0.002
		chest width	33.73 ± 2.63${}^{a}$ (n = 66)	33.15 ± 2.39${}^{\mathrm{ab}}$ (n = 123)	32.60 ± 1.76${}^{b}$ (n = 55)	0.009
		chest depth	55.93 ± 3.97${}^{a}$ (n = 66)	54.42 ± 3.31${}^{b}$ (n = 123)	54.23 ± 3.09${}^{b}$ (n = 55)	0.007
		rump length	46.30 ± 4.17${}^{a}$ (n = 66)	44.98 ± 5.55${}^{\mathrm{ab}}$ (n = 123)	43.92 ± 4.02${}^{b}$ (n = 55)	0.008
		rump width	41.45 ± 3.27${}^{a}$ (n = 66)	40.04 ± 3.51${}^{b}$ (n = 123)	39.47 ± 2.52${}^{b}$ (n = 55)	0.002
		rump height	140.58 ± 6.28${}^{a}$ (n = 66)	138.38 ± 6.36${}^{b}$ (n = 123)	138.43 ± 6.06${}^{\mathrm{ab}}$ (n = 55)	0.023
		body weight	305.85 ± 43.64${}^{a}$ (n = 40)	274.49 ± 52.50${}^{b}$ (n = 74)	284.86 ± 34.82${}^{\mathrm{ab}}$ (n = 22)	0.004
*ACSL1-3*	Female	withers height	139.07 ± 12.92${}^{a}$ (n = 14)	133.16 ± 6.34${}^{b}$ (n = 86)	135.45 ± 6.02${}^{a}$ (n = 105)	0.004
		chest depth	55.89 ± 3.72${}^{\mathrm{ab}}$ (n = 14)	55.17 ± 3.38${}^{b}$ (n = 86)	56.25 ± 3.03${}^{a}$ (n = 105)	0.022
		rump width	44.46 ± 5.17${}^{a}$ (n = 14)	42.19 ± 3.34${}^{b}$ (n = 86)	43.28 ± 3.15${}^{a}$ (n = 105)	0.019
	Male	rump width	42.70 ± 3.83${}^{a}$ (n = 10)	40.68 ± 3.57${}^{\mathrm{ab}}$ (n = 96)	39.85 ± 2.99${}^{b}$ (n = 105)	0.008
		body weight	305.58 ± 42.06 (n = 6)	280.43 ± 57.99 (n = 59)	287.81 ± 41.11 (n = 71)	0.413

Values with different superscripts within the same column differ significantly at P<0.05 (a, b).

**Table 5:** Association analysis between combined haplotypes of the *ACSL1* gene and growth traits in the Dezhou donkey.

Gender	Diplotypes	Observed genotypes (mean ± SE)							
		withers height	body length	chest circumference	chest width	chest depth	rump length	rump width	rump height
Female	Hap1Hap1 (n=3)	129.17 ± 8.13${}^{\mathrm{bc}}$	128.33 ± 5.85${}^{b}$	143.50 ± 2.17	31.00 ± 1.32	54.67 ± 3.06	41.00 ± 1.00${}^{b}$	41.17 ± 2.36	130.50 ± 8.26${}^{b}$
	Hap3Hap3 (n=4)	141.00 ± 4.74${}^{a}$	140.50 ± 8.10${}^{a}$	152.38 ± 5.71	31.25 ± 1.33	57.63 ± 2.06	43.50 ± 1.29${}^{\mathrm{ab}}$	43.00 ± 2.48	142.60 ± 3.64${}^{a}$
	Hap4Hap4 (n=4)	127.75 ± 8.26${}^{c}$	129.75 ± 7.93${}^{b}$	142.75 ± 8.42${}^{b}$	30.25 ± 0.95	54.75 ± 4.19	42.25 ± 3.30${}^{b}$	41.13 ± 1.75	130.75 ± 9.91${}^{b}$
	Hap5Hap5 (n=20)	134.18 ± 4.33${}^{b}$	135.40 ± 4.31${}^{\mathrm{ab}}$	151.75 ± 5.83${}^{a}$	31.95 ± 1.79	56.50 ± 1.86	43.20 ± 2.26${}^{b}$	43.85 ± 2.77	135.90 ± 4.93${}^{\mathrm{ab}}$
	Hap6Hap6 (n=5)	140.40 ± 7.60${}^{a}$	141.00 ± 10.22${}^{a}$	149.90 ± 11.07	32.70 ± 4.49	57.80 ± 4.08	46.80 ± 4.71${}^{a}$	43.50 ± 3.64	141.60 ± 8.14${}^{a}$
	P value	0.004	0.020	0.088	0.496	0.299	0.044	0.307	0.020
Male	Hap2Hap2 (n=3)	136.67 ± 2.52	134.17 ± 1.04	148.00 ± 10.14	35.50 ± 2.29${}^{a}$	57.00 ± 3.50${}^{\mathrm{ab}}$	46.33 ± 5.86	41.00 ± 1.73${}^{\mathrm{bc}}$	141.33 ± 3.06
	Hap3Hap3 (n=3)	140.67 ± 4.39${}^{a}$	142.50 ± 6.54	156.33 ± 3.78${}^{a}$	35.83 ± 2.02${}^{a}$	59.83 ± 6.33${}^{a}$	48.17 ± 2.47	44.17 ± 2.47${}^{\mathrm{ab}}$	143.00 ± 1.00
	Hap4Hap4 (n=4)	137.87 ± 2.39	136.75 ± 2.87	147.13 ± 6.88	33.63 ± 0.95${}^{\mathrm{ab}}$	54.13 ± 2.39${}^{b}$	45.25 ± 3.50	39.75 ± 1.84${}^{c}$	140.63 ± 3.19
	Hap5Hap5 (n=38)	135.53 ± 6.38${}^{b}$	133.11 ± 7.38	147.07 ± 7.80${}^{b}$	32.48 ± 1.83${}^{b}$	54.30 ± 3.28${}^{b}$	44.07 ± 4.28	39.42 ± 2.48${}^{c}$	138.08 ± 6.33
	Hap6Hap6 (n=5)	135.90 ± 10.87	138.70 ± 9.67	155.10 ± 6.27${}^{a}$	34.90 ± 2.07${}^{a}$	56.20 ± 3.95${}^{\mathrm{ab}}$	47.90 ± 2.51	45.00 ± 3.81${}^{a}$	139.60 ± 9.53
	P value	0.721	0.145	0.095	0.001	0.074	0.187	0.000	0.607

All individuals (n<3) are ignored in the analysis. Values with different superscripts within the same column differ significantly at P<0.05 (a, b, c).

## 4 Discussion

The donkey industry has been vigorously developed in many areas of northern China, but the genetic mechanism of growth and development traits of the Dezhou donkey have rarely been studied. Therefore, it is necessary to analyze the genetic variation of genes related to growth and development of the Dezhou donkey.

When analyzing the differences in expression levels of *ACSL* subtypes in rat myocardium, only the expression level of the *ACSL1* gene gradually increased after birth and reached the highest value in adulthood. Analysis of the expression level of the *ACSL1* gene in different tissues of Dezhou donkeys at different periods showed that the expression level of the *ACSL1* gene in seven tissues was higher in adult donkeys than in young ones, indicating that the expression level of the *ACSL1* gene in Dezhou donkeys was consistent with previous research results. In addition, the expression level of the *ACSL1* gene was higher in the liver of Dezhou donkeys, which was consistent with the results of the study on the expression level of cattle and sheep liver tissues (Cao, 2016; Zhao, 2016).

In the present study, four indels' polymorphic loci were found on the *ACSL1* gene of Dezhou donkeys, all of which located in the intron region. Although introns are non-coding protein sequences, they are involved in the regulation of gene expression. For example, when studying the dwarf phenotype of the Guizhou pony, it was found that the genetic variation of 5${}^{\prime }$ UTR (untranslational region) of the *IGF1* gene resulted in one fewer *cis*-acting element/transcription factor binding site for the Guizhou pony than for the Ili horse, which may affect the transcription efficiency of the *IGF1* gene (Wang et al., 2016). The rs1, rs2, rs4 and rs5 loci found in the *NCAPG* gene, the rs008 loci found in the *DCAF16* gene and the rs7 loci found in the *TBX3* gene all had significant effects on the early growth traits of the Dezhou donkey, which could be used for the molecular marker selection of the Dezhou donkey; especially the rs008 site has a very significant effect on body weight and withers height (Hou, 2019). So far, genetic polymorphism of the *ACSL1* gene has not been reported in Dezhou donkeys. Therefore, this study is of great significance for the exploration of genetic variation of the *ACSL1* gene in Dezhou donkeys and can provide some references for molecular breeding of Dezhou donkeys.

In this research, *ACSL1-4* was not in HWE in female donkeys. It could be that the sample size of our research was limited or that the female donkey experienced intense selection pressure, which causes the loss of certain alleles (Huang et al., 2013). The genetic diversity and genetic potential of the Dezhou donkey population can be tested by estimating the genetic parameters $H_{o}$, $H_{e}$, $N_{e}$ and PIC of the male and female populations. From the genetic information parameters in Table 2, it can be seen that the $H_{o}$, $N_{e}$ and PIC of *ACSL1-1* show lower values in the genes, indicating that there are lower levels of variation and weaker selection potential in the Dezhou donkey. Genetic diversity is essential for species preservation and improvement of potential production in selecting breeds (Huang et al., 2011). At the same time, our results showed that the linkage disequilibrium analysis of the four mutant sites was essentially the same in both the female and male populations of the Dezhou donkey. There are different degrees of linkage disequilibrium between different loci as shown in Fig. 5. Linkage disequilibrium plays a vital role in identifying the association between genetic markers and functional genes (Hou, 2019). As can be seen in Table 3, the haplotype (frequency >0.05) constructed was consistent in both female and male donkeys. Compared with single-mutation-site analysis, haplotype construction can improve the accuracy of association analysis and is a more reliable and effective molecular marker (Horne and Camp, 2004; Rodriguez et al., 2006)

A single-site association analysis found that the deletion or insertion of the four mutation sites of the *ACSL1* gene was indeed related to three important indicators of the growth traits of the female donkey – including withers height, body length and rump width – as well as with male withers height, body length, rump width and body weight, which suggests that *ACSL1* gene mutations might be related to the rump width of the Dezhou donkey. In the field of molecular marker research, there are many examples of correlation analysis of livestock candidate genes with growth traits. For example, studies have shown that there is a SNP (single-nucleotide polymorphism) site in the 3${}^{\prime }$ flanking region of the *IGF1* gene in Mongolian horses, which significantly affects the Mongolian horses in terms of withers height, body length, chest circumference and cannon circumference (Meng, 2009). Studies on the donkey *NR6A1* gene have shown that a 13 bp deletion found in the intron is related to the body features of Dezhou donkeys and Guanzhong donkeys, especially the withers height and body length (Fang et al., 2019). So finding loci related to growth traits will provide a theoretical reference for molecular breeding of Dezhou donkeys.

By combining haplotype combinations with single locus effects, the effects of genetic variation can be more easily demonstrated (Gui et al., 2015). It can be seen from Tables 4 and 5 that the results of haplotype combination analysis and unit point analysis of the mutation site of the *ACSL1* gene are consistent. In other words, the haplotype combinations Hap3Hap3 (II-II-DD-DD) and Hap6Hap6 (DD-II-II-II) were superior to other haplotype combinations in the *ACSL1* gene, while the unit points *ACSL1-1* (DD), *ACSL1-2* (II) and *ACSL1-3* (II) were superior in growth traits, which was consistent with haplotype Hap6Hap6 (DD-II-II-II). These results suggest that haplotype combinations of Hap6Hap6 (DD-II-II-II) in female and male donkeys can be used as molecular markers of the combined genotypes for future breeding selection of Dezhou donkeys.

## 5 Conclusion

The purpose of this study was to investigate the expression level and genetic variation of the *ACSL1* gene of the Dezhou donkey and its effect on growth traits. The results showed that the *ACSL1* gene is regularly expressed in heart, liver, spleen, lung, renal, gastric and muscle tissues of young and adult Dezhou donkeys. By analyzing the genetic parameters, linkage disequilibrium and haplotype of the four mutation loci of the *ACSL1* gene in the Dezhou donkey, it was found that there is little difference between the mutation loci in the female and male donkey populations. The results of the association analysis of the genotype amd haplotype combination of the *ACSL1* gene mutation site with the growth traits of the Dezhou donkey are basically consistent, which proves the reliability of the two. This lays a theoretical foundation for the follow-up study on the mechanism of related genes in the growth and development of the Dezhou donkey.

## Data Availability

The data are currently not publicly available. Please contact the corresponding author for further information.
